# In Silico Comparative Analysis of the Plant Growth Regulators Forchlorfenuron (CPPU) and Strigol (STG) Interacting with the Gibberellin Biosynthetic Enzyme GA3Ox2 and the Auxin Signaling Protein Receptor IAA7

**DOI:** 10.3390/ijms27072925

**Published:** 2026-03-24

**Authors:** Giovanny Hernández Montaño, Dulce Estefanía Nicolas Álvarez, Silvia Patricia Paredes Carrera, Benjamín Iván Romero De La Rosa, Jorge Alberto Mendoza Pérez

**Affiliations:** 1 Laboratorio de Tecnologías Limpias de Sistemas Ambientales, Escuela Nacional de Ciencias Biológicas (ENCB), Instituto Politécnico Nacional, Av. Wilfrido Massieu Esq. Cda. Miguel Stampa S/N, Gustavo A. Madero, Ciudad de Mexico 07738, Mexico; ghernandezm2100@alumno.ipn.mx; 2 Departamento de Fisiología, Escuela Nacional de Ciencias Biológicas (ENCB), Instituto Politécnico Nacional, Av. Wilfrido Massieu Esq. Cda. Miguel Stampa S/N, Gustavo A. Madero, Ciudad de Mexico 07738, Mexico; dnicolas@ipn.mx; 3 Laboratorio Nanomateriales Sustentables, Escuela Superior de Ingeniería Química e Industrias Extractivas, Instituto Politécnico Nacional, Unidad Profesional Adolfo López Mateos, Mexico City 07708, Mexico; silviappcar@gmail.com

**Keywords:** strigol (STG), forchlorfenuron (CPPU), molecular docking (MD), plant growth regulation (PGR), key protein receptors (KPR), in silico modeling, strigolactones

## Abstract

Plant growth regulation is orchestrated by complex hormonal networks involving gibberellin and auxin signaling pathways. In this study, a comprehensive in silico approach was employed to comparatively evaluate the plant growth regulators (PGRs) forchlorfenuron (CPPU) and strigol (STG) against two key proteins from Arabidopsis thaliana: Gibberellin 3-beta-dioxygenase 2 (GA3Ox2), a rate-limiting enzyme in the biosynthesis of bioactive gibberellins, and the auxin signaling repressor IAA7. These targets were specifically selected because they represent critical regulatory nodes in two major hormonal pathways controlling plant growth: GA3Ox2 governs the final steps of gibberellin activation, while IAA7 modulates auxin-responsive gene expression through its interaction with Auxin Response Factors. Therefore, their combined analysis enables the evaluation of potential regulatory effects of PGRs on both gibberellin biosynthesis and auxin-mediated transcriptional control. Molecular docking analyses revealed that both ligands exhibited higher binding affinity toward GA3Ox2 than IAA7, with STG showing slightly more favorable binding energies (−7.91 kcal/mol for GA3Ox2 and −5.43 kcal/mol for IAA7) compared to CPPU (−7.18 and −4.79 kcal/mol, respectively). These results suggest a structural preference of both PGRs toward the gibberellin biosynthetic pathway. To further assess complex stability under near-physiological conditions, 100 ns molecular dynamics (MD) simulations were conducted using the CHARMM36m force field. Despite its slightly lower docking scores, CPPU demonstrated greater conformational stability, lower RMSD fluctuations, and more persistent hydrogen bonding patterns, particularly in complexes with IAA7. In contrast, STG induced more pronounced conformational rearrangements, although it promoted slightly more compact protein conformations in certain systems. Fourier-transform infrared (FTIR) spectroscopy supported the computational findings by confirming the presence of key functional groups responsible for hydrogen bonding and hydrophobic interactions. Collectively, the results indicate that although STG exhibits higher initial binding affinity, CPPU forms more dynamically stable complexes with both proteins. These findings suggest that CPPU may represent a more robust candidate for sustained modulation of auxin and gibberellin signaling pathways in plant growth regulation.

## 1. Introduction

Plant growth and development are regulated by a complex network of phytohormones that act in a coordinated manner to modulate processes such as cell elongation, branching, differentiation, and stress response [1]. Among these, gibberellins play a central role in regulating vegetative growth, particularly through key enzymes such as GA3-oxidase 2 (GA3Ox2), responsible for converting precursors into biologically active forms of gibberellins [2]. Likewise, within auxin signaling, the IAA7 protein, a member of the Aux/IAA family, acts as a key negative regulator of growth by interacting with Auxin Response Factors (ARFs) [3] and controlling auxin-dependent gene expression [4]. Its structural stability and conformational dynamics determine the intensity of the auxin response [5,6,7], making it a relevant molecular target for evaluating compounds with potential to modulate plant growth. GA3Ox2 and IAA7 were specifically selected as targets in this study due to their central roles in hormonal regulation, their well-characterized structures, and evidence of cross-talk between gibberellin and auxin pathways, which suggests that compounds interacting with both targets may influence plant growth through coordinated hormonal modulation. This allows us to explicitly explore whether CPPU and STG may exert effects across multiple signaling pathways, providing insights into potential diafonic interactions in plant growth regulation.

In recent years, interest in identifying and optimizing high-potential plant growth regulators has increased considerably in both agriculture and biotechnology [8]. Within this context, strigolactones, represented by compounds such as estrigol (STG), have been extensively studied for their role in regulating plant architecture, inhibiting lateral shoot growth, and rhizosphere signaling [9]. Simultaneously, synthetic regulators such as forchlorfenuron (CPPU), a cytokinin analog, have demonstrated significant effects on cell division, fruit size, and agricultural yield [10]. Understanding the interactions of these compounds with key hormonal proteins can provide practical insights for the rational design of regulators aimed at improving crop yield, controlling plant architecture, and optimizing stress responses in agricultural systems.

Despite abundant experimental evidence on the physiological effects of both phytohormones (STG and CPPU), the molecular mechanisms underlying their interaction with specific targets of metabolism and hormonal signaling have not yet been fully elucidated [11]. In particular, the comparative evaluation of their potential interaction not only with GA3Ox2 (a key enzyme in gibberellin biosynthesis) but also with central regulators of the auxin pathway, such as the IAA7 protein [4], could provide relevant information on their potential to modulate both gibberellin biosynthesis and the intensity of the auxin response [12]. Since IAA7 acts as a transcriptional repressor whose structural stability conditions auxin-dependent gene activation [3], its analysis as a molecular target would allow for the exploration of possible indirect mechanisms of plant growth regulation through the modulation of hormonal balance.

In this regard, in silico tools, such as molecular docking [13] and molecular dynamics (MD) [14], allow for the exploration of the binding affinity, structural stability, and conformational behavior of ligand–protein complexes under simulated conditions close to the biological environment [13]. These approaches offer a robust platform for the preliminary analysis of compounds with regulatory potential, reducing costs and experimental time [15].

This study aims to perform a comparative analysis of forchlorfenuron (CPPU) and strigol (STG) using molecular docking studies and molecular dynamics simulations against the GA3Ox2 enzyme and the IAA7 protein [16], in order to evaluate their affinity, structural stability, and potential differences in their dynamic behavior. The results obtained seek to provide computational evidence that contributes to understanding their potential as plant growth regulators (PGRs) and their practical applications in agriculture and crop improvement [17].

## 2. Results

**Figure 1:**
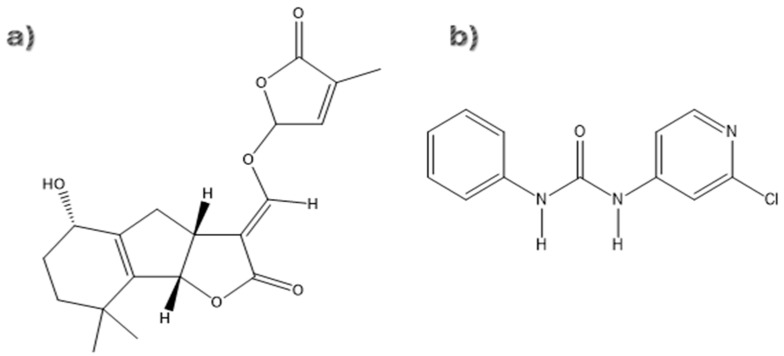
Structures of the evaluated ligands: (**a**) Strigol (STG), (**b**) Forchlorfenuron (CPPU).

In this study, a simulation model was developed to predict the interaction of the plant growth regulators (PGRs) CPPU and STG with gibberellin receptors (GA3Ox2) and auxin receptors (IAA7) as candidate PGRs. The aim was to compare these two PGRs, as both CPPU and STG have shown high potential as plant growth regulators in previous work. This was achieved using an integrative in silico strategy that combines molecular docking and molecular dynamics. Finally, the results obtained by FTIR spectroscopy were analyzed to identify the functional groups of CPPU and STG molecules and correlate them with the predicted interactions (Figure 1).

### 2.1. Molecular Docking Results

Molecular docking simulations provided an approximation of the binding affinity energy and the interacting amino acids involved between forchlorfenuron and strigol, each with two key plant receptors: GA3Ox2 and IAA7.

#### 2.1.1. Binding Between CPPU and IAA7 and GA3Ox2

Statistical analysis showed that CPPU has a binding energy of −4.79 kcal/mol with IAA7, interacting with ARG12, LYS13, VAL8, and ASN10. With GA3Ox2, it showed a higher affinity (−7.18 kcal/mol) [18,19], interacting with ASN210, ARG295, ASP229, HIS227, ILE119, LEU224, PHE301, SER297, TYR212, and VAL236.

**Figure 2:**
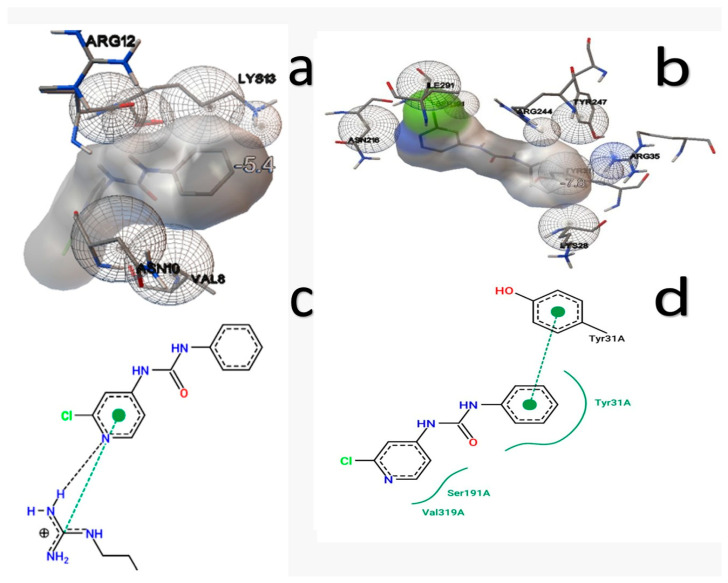
Docking analysis with the IAA7 and GA3Ox2 receptors. Interaction spheres with (**a**) IAA7-CPPU and (**b**) GA3Ox2-CPPU, and 2D interaction maps with (**c**) IAA7-CPPU and (**d**) GA3Ox2-CPPU.

The 2D interaction diagrams for IAA7 illustrate hydrogen bonds between the chlorinated heterocyclic ring of CPPU and the amino group of the receptor. For GA3Ox2 [20], electrostatic interaction was observed between the benzene ring and Tyr31A, along with hydrophobic interactions involving Tyr31A, Ser191A, and Val319A (Figure 2).

#### 2.1.2. Binding of STG to IAA7 and GA3Ox2

STG exhibited a binding energy of −5.43 kcal/mol with IAA7, interacting with ARG12, ASN10, GLY4, LYS13, VAL3, and VAL8. With GA3Ox2, it exhibited higher affinity (−7.91 kcal/mol), interacting with ARG132, ARG133, THR129, and VAL53.

**Figure 3:**
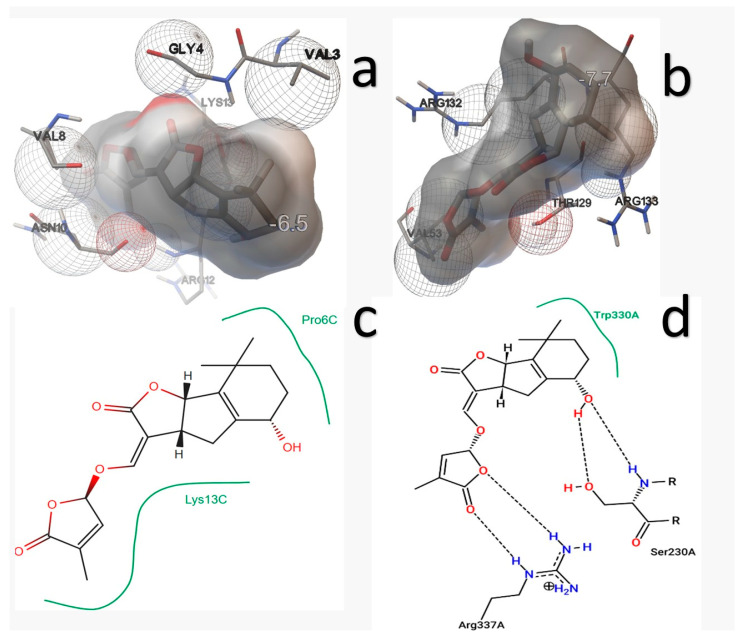
Docking analysis with the IAA7 and GA3Ox2 receptors. Interaction spheres with (**a**) IAA7-STG and (**b**) GA3Ox2-STG, and 2D interaction maps with (**c**) IAA7-STG and (**d**) GA3Ox2-STG.

The 2D interaction diagrams show hydrophobic interactions between STG and Pro6C and Lys13C in IAA7. For GA3Ox2, hydrophobic interaction was observed between the STG ring and Trp330A, while the hydroxyl group formed two hydrogen bonds with Ser230A. Additionally, the ring oxygen and carbonyl oxygen formed hydrogen bonds with Arg337A (Figure 3).

Table 1 summarizes the coupling scores and interacting residues for both receptors. In both ligands, affinity for GA3Ox2 was greater than for IAA7, with an energy difference of ~2.4–2.5 kcal/mol.

**Figure 4:**
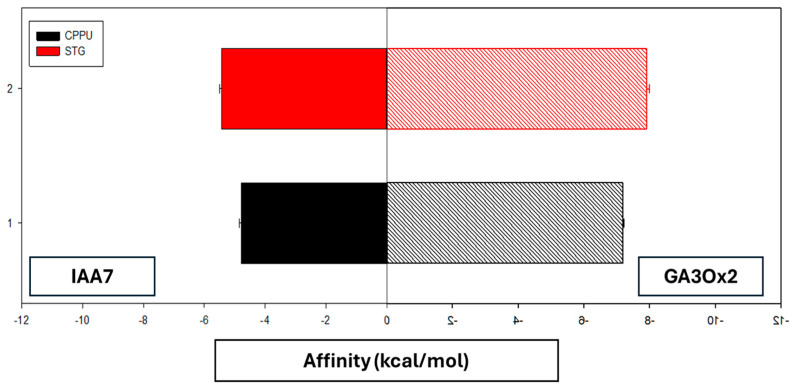
Binding affinity energies (mean ± SE) of each ligand–receptor interaction obtained through molecular docking simulations. Solid bars represent interactions of IAA7 with CPPU (black) and STG (red). Hatched bars represent interactions of GA3Ox2 with the same PGRs. More negative values indicate a stronger binding affinity.

Binding affinity energies (mean ± SE) of each ligand–receptor interaction obtained through molecular docking simulations are shown in Figure 4.

When comparing ligands, STG showed slightly more favorable energies than CPPU for both receptors (≈0.6 kcal/mol improvement for IAA7 and ≈0.7 kcal/mol for GA3Ox2). Binding energies for IAA7 (−4.79 and −5.43 kcal/mol) were moderate, whereas GA3Ox2 interactions (−7.18 and −7.91 kcal/mol) were within a range associated with more stable protein–ligand interactions.

### 2.2. Molecular Dynamics Results

To evaluate conformational stability, 100 ns molecular dynamics simulations were performed for CPPU–IAA7, CPPU–GA3Ox2, STG–IAA7, and STG–GA3Ox2 complexes using GROMACS v2022.4 and the CHARMM36m force field under near-physiological conditions.

#### 2.2.1. CPPU and STG with IAA7

**Figure 5:**
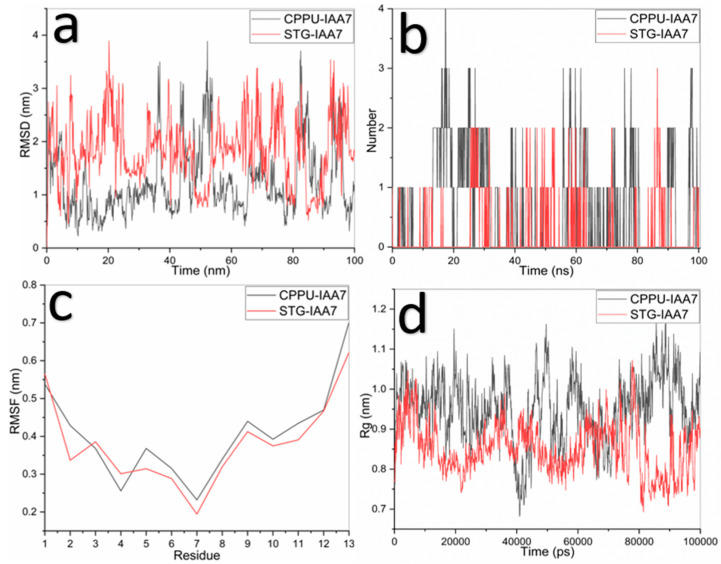
Molecular dynamics results for CPPU (black line) and STG (red line) with IAA7 ligand; (**a**) RMSD LIG after alignment to the backbone, (**b**) hydrogen bonds, (**c**) RMS fluctuation, and (**d**) radius of gyration.

The CPPU–IAA7 complex maintained RMSD values around 0.8–1.2 nm with moderate fluctuations, while STG–IAA7 showed larger deviations (1.5–2.5 nm, peaks >3.0 nm). CPPU established 1–2 persistent hydrogen bonds, occasionally reaching 3, whereas STG predominantly showed 0–1 hydrogen bonds. RMSF analysis revealed similar patterns, though STG displayed higher fluctuations at terminal residues. STG induced slightly greater receptor compaction (Rg ~0.78–0.90 nm) compared to CPPU (~0.95–1.05 nm). Figure 5 shows RMSD, hydrogen bonds, RMSF, and Rg analyses (Figure 5).

#### 2.2.2. CPPU and STG with GA3Ox2

**Figure 6:**
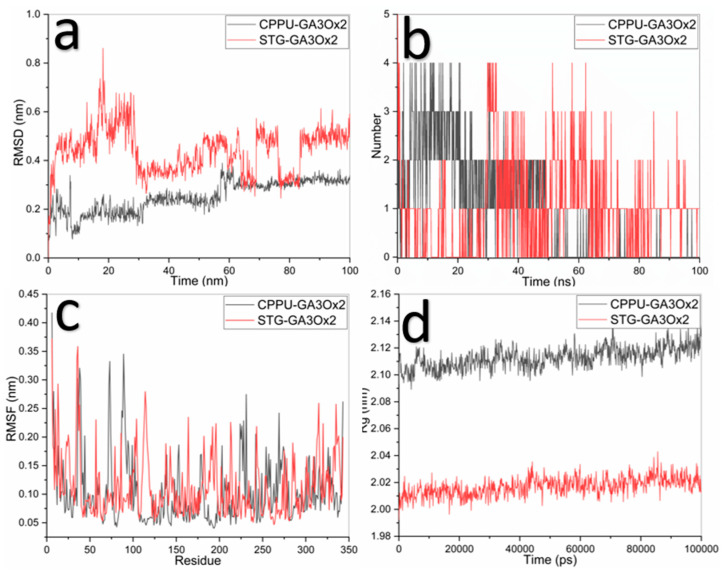
Molecular dynamics results for CPPU (black line) and STG (red line) with GA3Ox2 ligand; (**a**) RMSD, (**b**) Hydrogen bonds, (**c**) RMS fluctuation, and (**d**) Radius of gyration.

The CPPU–GA3Ox2 complex stabilized around 0.2–0.4 nm RMSD after 20 ns, while STG–GA3Ox2 initially showed higher RMSD (~0.5–0.8 nm) that later stabilized (~0.3–0.5 nm). CPPU maintained 2–3 hydrogen bonds in the first half of the simulation, whereas STG maintained 1–2 intermittent bonds. Both complexes showed low RMSF fluctuations (<0.4 nm). CPPU–GA3Ox2 maintained Rg values of 2.10–2.14 nm, while STG–GA3Ox2 exhibited slightly lower Rg (2.00–2.04 nm) (Figure 6).

#### 2.2.3. FTIR Spectroscopy

CPPU exhibited a band around 3400 cm^−1^ corresponding to N–H stretching [21], a peak near 1700 cm^−1^ associated with C=O, and bands at ~1600 and ~1100 cm^−1^ assigned to C=C and C–Cl bonds [17].

**Figure 7:**
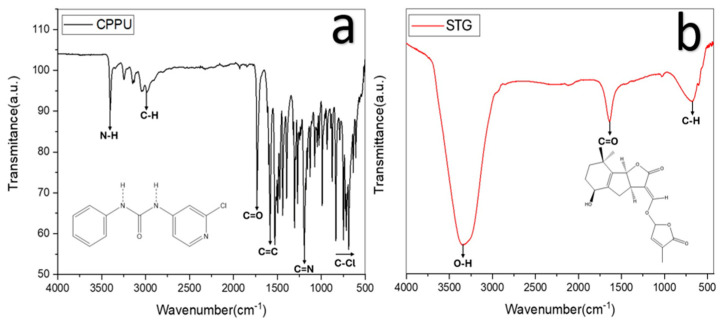
Fourier transform infrared (FTIR) spectroscopy: (**a**) forchlorfenuron (CPPU), (**b**) strigol (STG).

STG showed a strong O–H stretching band near 3400 cm^−1^, a carbonyl band around 1700 cm^−1^, and C–H vibrations near 2900 cm^−1^ [22]. FTIR spectra (Figure 7) revealed characteristic functional groups in both molecules that are capable of participating in hydrogen bonding or hydrophobic interactions. While these spectra do not directly measure protein–ligand interactions, they complement the in silico docking and molecular dynamics results by confirming the presence of chemical features relevant for potential binding interactions.

## 3. Discussion

The comparative docking analysis demonstrated a consistent preference of both CPPU and STG for the GA3Ox2 receptor over IAA7. The ~2.4–2.5 kcal/mol energy difference suggests greater stability and a higher probability of ligand retention within the GA3Ox2 binding pocket. This pattern indicates a possible structural preference for proteins associated with the gibberellin pathway.

Although STG showed slightly more favorable docking energies than CPPU for both receptors, the molecular dynamics simulations revealed important differences in temporal stability. In the IAA7 complexes, CPPU promoted more consistent conformational stabilization, with lower RMSD fluctuations and more persistent hydrogen bonds compared to STG. This suggests that, despite STG’s slightly better docking score, CPPU may establish more stable interactions with IAA7 under dynamic conditions.

For GA3Ox2, both ligands formed stable complexes; however, CPPU exhibited faster stabilization, lower RMSD values, and a higher average number of hydrogen bonds during the simulation. STG induced a slightly more compact receptor conformation but required a longer conformational adjustment phase. These findings complement the docking results and reinforce the relevance of GA3Ox2 as a primary interaction target.

The FTIR analysis confirmed the presence of functional groups in CPPU and STG capable of forming hydrogen bonds and hydrophobic interactions (N–H in CPPU and O–H in STG, as well as carbonyl, aromatic, and halogen groups). Although these spectra do not directly indicate protein–ligand interactions, they support the chemical features predicted to participate in binding, complementing the computational docking and molecular dynamics findings.

Overall, the combined docking, molecular dynamics, and FTIR results indicate that both CPPU and STG exhibit stronger and more stable interactions with GA3Ox2 than with IAA7. While STG shows slightly more favorable binding energies, CPPU demonstrates greater dynamic stability in certain complexes. These findings suggest that modulation of GA3Ox2 may represent a key component in the molecular mechanism of action of both regulators, a hypothesis that warrants further experimental validation.

Despite these insights, several questions remain open. Experimental confirmation in planta is necessary to verify the predicted ligand–receptor interactions and their effect on plant growth. Additionally, exploring other receptors and signaling components involved in gibberellin and auxin pathways could provide a more comprehensive understanding of the regulatory network. Future studies may also investigate the design of optimized analogs based on CPPU and STG structures to enhance efficacy and specificity in plant growth modulation. Addressing these aspects will be essential to translate computational findings into practical agricultural applications and to fully elucidate the molecular mechanisms underlying the action of these plant growth regulators.

## 4. Materials and Methods

The present work employed an in silico computational strategy to assess and compare the molecular interactions and conformational stability of forchlorfenuron (CPPU) and strigol (STG). Both compounds were evaluated against two essential proteins associated with plant growth regulation, GA3Ox2 and IAA7. The methodological framework comprised the structural preparation of ligands and proteins, molecular docking analyses, molecular dynamics simulations, and functional group characterization through Fourier transform infrared (FTIR) spectroscopy, Infrared spectra were recorded with a PerkinElmer Frontier spectrometer, Macedonio Alcala, CDMX, Mexico in transmission mode, covering the range from 400 to 4000 cm^−1^. All computational experiments were conducted with 100 independent runs per ligand–protein system to calculate average binding affinities and ensure statistical reliability. Ligand conformations for docking were randomly sampled to minimize selection bias.

### 4.1. Software and Online Tools

The computational workflow incorporated several specialized software packages and online platforms. Avogadro (version 2.0 (2022) [23] was applied for ligand geometry optimization (https://avogadro.cc), while ChemDraw [18] (https://www.perkinelmer.com) and Chem3D [19] (https://www.perkinelmer.com) were used for molecular design. Structural visualization and protein preparation were performed using UCSF Chimera [24] (https://www.cgl.ucsf.edu/chimera/). Molecular docking calculations and file processing were carried out with AutoDock Vina [15] (http://vina.scripps.edu) in combination with AutoDockTools [13] (http://autodock.scripps.edu). Molecular dynamics simulations were executed in GROMACS [24] (http://www.gromacs.org), and ligand topologies were generated through CHARMM-GUI [21] (http://www.charmm-gui.org). Binding pocket identification and interaction analysis were supported by ProteinPlus [25] (https://proteins.plus/) and DoGSiteScorer [26] (https://bio.tools/dogsitescorer).

### 4.2. Molecular Docking

Molecular docking analyses were conducted according to established computational procedures commonly applied in protein–ligand interaction research [27]. The crystallographic structures of the target proteins were obtained from the Protein Data Bank [28], specifically GA3Ox2 (PDB ID: 7EKD) and IAA7 (PDB ID: 2P1N) [29]. Each structure was preprocessed by removing crystallographic water molecules, co-crystallized ligands, and other heteroatoms to prevent steric artifacts, using UCSF Chimera [20] (https://www.cgl.ucsf.edu/chimera/).

The molecular models of forchlorfenuron (CPPU) and strigol (STG) were generated with ChemDraw [18] (https://www.perkinelmer.com) based on previously reported structural data [17]. All ligands were subsequently visualized and subjected to ground-state energy minimization employing Avogadro [23] (https://avogadro.cc) with the MMFF94 force field to ensure optimal geometries prior to docking.

Docking simulations were carried out using AutoDock Vina [15] (http://vina.scripps.edu), which utilizes a stochastic global optimization algorithm coupled with an empirical scoring function to predict binding poses and estimate binding free energies. The resulting complexes were inspected, processed, and converted into PDBQT format with AutoDockTools [13] (http://autodock.scripps.edu), where rotatable bonds and Gasteiger charges were assigned.

Search grids were defined around the experimentally determined active sites to confine conformational exploration to biologically relevant regions. For GA3Ox2, the grid center was established according to the coordinates of the co-crystallized ligand, using a grid box of 56.63 Å × 58.96 Å × 42.90 Å and a spacing of 1.0 Å. In the case of IAA7, the grid was centered at −27.46 Å × 21.33 Å × −34.24 Å with the same spacing parameter [30].

Docking calculations were repeated 100 times for each ligand–protein system to obtain average binding affinities, ensuring robust statistical reliability. The top-ranked binding modes were selected based on predicted binding energies and interaction patterns, including hydrogen bonding and hydrophobic contacts. This approach, widely implemented in plant hormone protein studies, offers a robust atomic-level approximation of ligand–protein affinity [31,32]. All computational tools employed in this workflow are publicly accessible.

### 4.3. Molecular Dynamics

Molecular dynamics (MD) simulations were conducted to investigate the structural stability and dynamic behavior of the receptor–ligand complexes under conditions approximating the physiological environment. All-atom simulations were carried out with GROMACS [20,32], which integrates Newton’s equations of motion to model the temporal evolution of atomic coordinates [32].

Ligand topologies were prepared using the Ligand Reader and Modeler module available through CHARMM-GUI [20], ensuring compatibility with the CHARMM36m force field, recognized for its robust parameterization of both proteins and small molecules [33]. Each protein–ligand complex was embedded in a cubic simulation box filled with TIP3P water molecules and neutralized by adding Na^+^ and Cl^−^ ions to reach a physiological ionic strength of 0.15 M [34].

Prior to production runs, systems underwent energy minimization using the steepest descent algorithm to remove unfavorable contacts. Equilibration was performed in two sequential phases: first, 100 ps under NVT conditions (constant number of particles, volume, and temperature) for 125,000 steps; second, 100 ps under NPT conditions (constant number of particles, pressure, and temperature). Temperature was controlled at 300 K using a velocity-rescaling thermostat, while pressure was maintained at 1 atm with the Parrinello–Rahman barostat. Subsequently, production MD simulations were executed for 100 ns with a 2 fs integration time step. Structural stability and interaction persistence were assessed by calculating root mean square deviation (RMSD) values and hydrogen bond distributions using the built-in analysis utilities of GROMACS [29,35].

## 5. Conclusions

In summary, the comprehensive comparison between CPPU and strigol (STG) as plant growth regulators reveals that, although both ligands exhibit favorable affinities for the IAA7 and GA3Ox2 receptors, STG consistently displays more negative docking energies, especially with GA3Ox2, indicating a higher initial affinity. However, molecular dynamics studies show that CPPU induces greater structural stability and hydrogen bond persistence, particularly in the complex with IAA7, where it exhibits lower conformational deviations (RMSDs) and a more sustained polar interaction, suggesting a longer-lasting interaction under dynamic conditions. On the other hand, with GA3Ox2, CPPU also maintains superior conformational stability and a greater number of hydrophilic bonds throughout the simulation, while STG favors a slightly more compact conformation, but with lower overall dynamic stability. FTIR spectroscopy corroborates these differences, showing that CPPU has N–H and C=O functional groups capable of forming more stable hydrogen bonds, while STG stands out for its O–H group, which, although it promotes polar interactions, appears to generate a more fluctuating dynamic in the complex. Taken together, these results suggest that, although STG has a higher binding affinity according to docking, CPPU is the regulator that most likely ensures a more stable and prolonged interaction with both receptors, especially under dynamic physiological conditions. This could translate into more effective modulation of the auxin and gibberellin hormonal pathways. Therefore, CPPU emerges as the most robust candidate for practical applications in plant growth regulation.

## Figures and Tables

**Table 1:** Coupling scores and residue interactions for IAA7 and GA3Ox2.

Receptor	Ligand	Docking Score	Interaction Aminoacids
IAA7	CPPU	−4.79	**ARG12**, **LYS13**, **VAL8**, **ASN10**
	STG	−5.43	**ARG12**, **ASN10**, GLY4, **LYS13**, VAL3, **VAL8**
GA30X2	CPPU	−7.18	ASN210, ARG295, ASP229, HIS227, ILE119, LEU224, PHE301, SER297, TYR212, VAL236
	STG	−7.91	ARG132, ARG133, THR129, VAL53

## Data Availability

The original contributions presented in the studio are included in the article.

## Abbreviations

The following abbreviations are used in this manuscript:

CPPU	Forchlorfenuron
STG	Strigol
PGR	Plant Growth Regulator
KPR	Key Protein Receptor
